# New phosphine-diamine and phosphine-amino-alcohol tridentate ligands for ruthenium catalysed enantioselective hydrogenation of ketones and a concise lactone synthesis enabled by asymmetric reduction of cyano-ketones

**DOI:** 10.1186/1752-153X-6-151

**Published:** 2012-12-10

**Authors:** José A Fuentes, Scott D Phillips, Matthew L Clarke

**Affiliations:** 1 School of Chemistry, University of St Andrews, EaStCHEM, St Andrews, Fife KY16 9ST, UK

**Keywords:** Hydrogenation, Homogeneous catalysis, Keto-nitriles, P,N,O ligands, P,N,N ligands, Asymmetric synthesis, Chiral alcohols, Organocatalysis, Michael addition, Acrylonitrile

## Abstract

Enantioselective hydrogenation of ketones is a key reaction in organic chemistry. In the past, we have attempted to deal with some unsolved challenges in this arena by introducing chiral tridentate phosphine-diamine/Ru catalysts. New catalysts and new applications are presented here, including the synthesis of phosphine-amino-alcohol *P,N,OH* ligands derived from (*R,S*)-1-amino-2-indanol, (*S,S*)-1-amino-2-indanol and a new chiral *P,N,N* ligand derived from (*R,R*)-1,2-diphenylethylenediamine. Ruthenium pre-catalysts of type [RuCl_2_(L)(DMSO)] were isolated and then examined in the hydrogenation of ketones. While the new *P,N,OH* ligand based catalysts are poor, the new *P,N,N* system gives up to 98% e.e. on substrates that do not react at all with most catalysts. A preliminary attempt at realising a new delta lactone synthesis by organocatalytic Michael addition between acetophenone and acrylonitrile, followed by asymmetric hydrogenation of the nitrile functionalised ketone is challenging in part due to the Michael addition chemistry, but also since Noyori pressure hydrogenation catalysts gave massively reduced reactivity relative to their performance for other acetophenone derivatives. The Ru phosphine-diamine system allowed quantitative conversion and around 50% e.e. The product can be converted into a delta lactone by treatment with KOH with complete retention of enantiomeric excess. This approach potentially offers access to this class of chiral molecules in three steps from the extremely cheap building blocks acrylonitrile and methyl-ketones; we encourage researchers to improve on our efforts in this potentially useful but currently flawed process.

## Findings

**Scheme 1:**
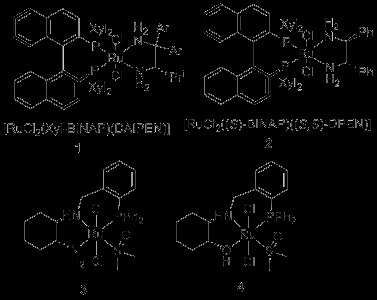
Hydrogenation catalysts.

Reduction of C = O and C = N double bonds using molecular hydrogen is a very important process, due to its low cost and complete atom efficiency [1]. Homogeneous hydrogenation of unfunctionalised ketones could not be carried out with sufficient efficiency or chemo-selectivity until the Noyori group’s pioneering research on ruthenium complexes containing both diphosphine and diamine ligands (e.g. **1** and **2,** Scheme 1) [2,3]. These catalysts give excellent results in the hydrogenation of a range of acetophenone derivatives, as have a number of structurally related catalysts [4,5]. However, [RuCl_2_(BINAP)(DAIPEN)] and related catalysts do have some important limitations, that have spurred significant interest in new catalyst development [6-21]. Given that so many drugs, agrochemicals, materials, and natural products can be disconnected back to enantiopure secondary alcohols, it is of significant importance to extend asymmetric hydrogenation chemistry such that it is effective for every major class of substrate. We have already reported on the reduction of some of these challenging substrates, namely bulky ketones [6,10,11], heterocycle appended (bulky) ketones [10], and certain esters [9]. To achieve this, we developed ruthenium complexes of chiral tridentate *P,N,N* and *P,N,OH* ligands (derived from cyclohexane diamine and aminocyclohexanol). These gave moderate to excellent enantioselectivity and high yields where there was a precedent for [RuCl_2_(BINAP)(DAIPEN)] giving very low yields. However, while these results are significant in developing *P,N,N* ligands for catalytic hydrogenation reactions, further improvements are needed for the catalysts to become industrially useful. In this paper, we report investigations into some alternative chiral backbones along with our initial attempt to apply these in a potentially concise asymmetric synthesis of delta lactones.

The original catalyst design presumed the primary amine terminus of the catalyst would bind ketones and activate hydrogen in the same manner as Noyori catalysts. However, our recent mechanistic studies showed that the secondary amine of catalyst **3** is influential in both the efficient activation of hydrogen, and (most likely) the control of selectivity in asymmetric hydrogenation [12]. Since the primary amine terminus in complex **3** seemed less important, we have reported a single example of a *P,N,O* ligand and its Ru complex, **4** that gave good results in the enantioselective hydrogenation of ketones [12] and good activity in the hydrogenation of aromatic esters [9]. An important extension was to investigate a different chiral backbone that had a strong heritage in asymmetric catalysis, 1-amino-2-indanol.

**Scheme 2:**
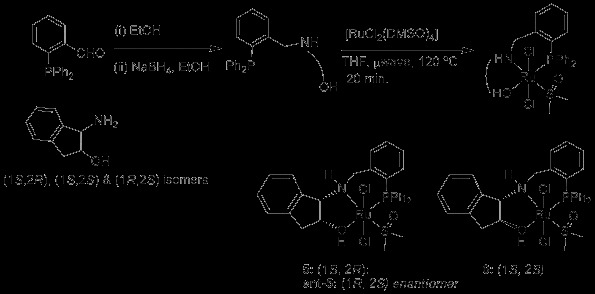
**Synthesis of *P,N,OH *ligands and their Ru(II) complexes.**

Both diastereomers of the phosphine-amino-alcohol ligands **5** and **6** were readily prepared by condensation with diphenylphosphino-benzaldehyde followed by reduction with NaBH_4_ with unoptimised yields of 75-97%. Reaction of phosphine-amino-alcohol ligands with [RuCl_2_(DMSO)_4_] gave, after purification, the diastereomeric complexes of formula [RuCl_2_(PNOH)(DMSO)], **5** and **6** in unoptimised yields of 67-78% (Scheme 2).

**Scheme 3:**
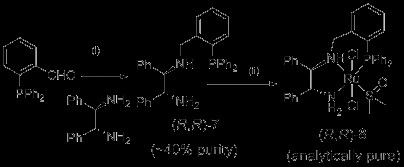
**Synthesis of DPEN-derived catalyst (*R*,*R*)-8. ***General conditions: *(i) (*R*,*R*)-(+)-1,2-diphenylethylenediamine (3 eq.), EtOH, 45°C → rt, 2 h; NaBH_4 _(4 eq.), EtOH, rt, 8 h; (ii) (*R*,*R*)-**7 **(1 eq.), [RuCl_2_(DMSO)_4_] (1 eq.), THF, μw, 120°C, 20 min.

With the new *P,N,OH* ligands in hand, we turned our attention to a new *P,N,N* system. There are of course many diamines known in the literature that might be used to make new derivatives of these catalysts. It was envisaged that through modification of the diamine component, the orientation of the mechanistically important N-H bond could be optimised to maximise catalyst activity and selectivity. Our previous research pointed towards tridentate ligands with relatively small N-N angles being most suitable and this prompted us to focus on a (*R*,*R*)-(+)-1,2-diphenylethylenediamine derived ligand. It is worth noting that the synthesis of ligands with a primary amine terminus often proves more difficult than the phosphine-amino-alcohol systems, or for that matter, most phosphine ligands. The synthesis of the ‘DPEN’-derived ligand threw up several synthetic difficulties preventing the isolation of pure ligand, but the crude ligand was converted into its Ru complex that was then purified using column chromatography and fully characterised (Scheme 3). This could then be compared with the parent catalyst **3**. On one occasion, this gave a 62% yield of complex **8**, with no purification of the crude ligand, but a more reproducible method to generate pure material was to semi-purify the ligand using column chromatography although this gave lower yields. In any case, our objective was to test a pure catalyst of this type and this route was sufficient to achieve that.

**Scheme 4:**
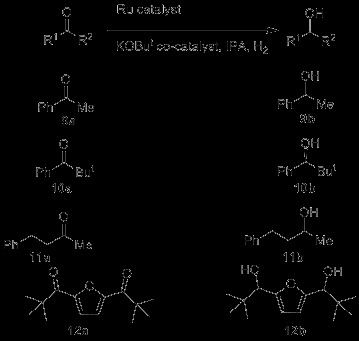
**Substrates and products of hydrogenation reaction reported in Tables**1**and **2.

Asymmetric hydrogenation of ketones, **9a-12a** (Scheme 4) was attempted using **5**, **ent-5** and **6** as the catalyst. Modest enantioselectivities were obtained in the hydrogenation of the easy substrate acetophenone, **9a** (Table 1, Entries 1–5). In contrast to the *P,N,N* systems, enantioselectivity improves as base/Ru is increased up to 10:1, although even in the best scenarios, only 51% e.e. for alcohol **9b** could be realised. The catalyst did not have enough activity to reduce the poorly reactive ketone, **10a** (Table 1, Entry 7). For comparison, some results using previously reported catalyst **4** are given. These show the hugely enhanced reactivity and selectivity with the bulky ketone **10a** using catalyst **4**, but we also highlight an issue of maintaining enantioselectivity when reducing catalyst loadings below 0.1%.

**Table 1:** Enantioselective hydrogenation of some ketones using Ru catalysts derived from phosphine-amino-alcohols

**Entry**^**[a]**^	**Cat**	**Ketone**	**[cat] mol%**	**[base] mol%**	**Conversion (yield) [%]**^**[b]**^	**e.e. [%]**^**[c]**^
1	**5**	**9a**	0.5	1	99	23 (*S*)
2	**6**	**9a**	0.5	1	99	27 (*R*)
3	**ent-5**	**9a**	0.5	1	99	29 (*R*)
4	**ent-5**	**9a**	0.5	2.5	99	43 (*R*)
5	**ent-5**	**9a**	0.5	5	98	51 (*R*)
6	**4**	**9a**	0.5	2.5	99 (99)	15 (*S*)
7	**5**	**10a**	0.5	1	26	4
8	**4**	**10a**	0.5	2.5	99 (99)	74 (*S*)
9^[d]^	**4**	**10a**	0.5	2.5	99	79 (*S*)
10^[e]^	**4**	**10a**	1	5	98	80 (*S*)
11	**4**	**10a**	0.09	5	45	3 (*S*)
12	**6**	**11a**	0.5	2.5	99 (97)	0

^[a] ^Unless otherwise indicated, reactions were carried out using 0.33 mmol mL^-1 ^of ketone at 50°C and 50 bar of hydrogen pressure with a 16 h reaction time using *t- *BuOK as base. ^[b] ^Conversions were determined by ^1^H NMR analysis of the crude reaction mixtures (all peaks were assigned). Yields are for pure alcohols after short-path silica gel chromatography. ^[c] ^The e.e. value was determined by chiral HPLC. ^[d] ^35°C. ^[e] ^3 h.

Table 2 reports the results for a small section of substrates that were hydrogenated with catalyst **8**.

**Table 2:** Enantioselective hydrogenation of some ketones using catalyst 8

**Entry**^***a***^	**Ketone**	**Catalyst**	**Time (h)**	**Conversion [%]**^***b***^	**e.e. [%]**^***c***^
1	**9a**	(*R,R*)-8 (0.5%)	16	>99	3
2	**10a**	(*R,R*)-8 (0.5%)	16	>99	80 (*S*)
3	**10a**	(*R,R*)-3 (0.5%)	16	>99	74 (S)
4	**10a**	(*R,R*)-8 (0.33%)	1	44	56 (*S*)
5	**10a**	(*S,S*)-3 (0.33%)	1	45	65 (*R*)
6	**11a**	(*R,R*)-8	16	>99	9
7	**12a**	(*R,R*)-8	16	>99	98^d^
8 ^e^	**12a**	(*R,R*)-3	16	>99	98^f^

^*a *^Reactions were carried out using 0.33 mmol mL^-1 ^of ketone, 0.5 mol% preformed catalyst (*R*,*R*)-**27 **and 1 mol% KO^*t *^Bu at 70°C and 50 bar of hydrogen pressure with a 16 h reaction time. ^*b *^Conversions were determined by ^1^H NMR analysis of the crude reaction mixtures (all peaks assigned). ^*c *^The e.e. value was determined by chiral HPLC (Chiralpak AD-H/Chiralcel OD-H column). The absolute configuration was determined to be *S *by comparison of optical rotation values with literature values. ^d ^((*R*,*R*) + (*S*,*S*)):*meso *3.1:1. ^e ^Reaction temperature of 50°C. ^f ^{(*R*,*R*) + (*S*,*S*)}:*meso *2.8:1.

Catalyst **8** was found to hydrogenate ketones quantitatively after 16 hours at 50°C and 50 bar hydrogen pressure (Table 3). Phenethyl alcohol was produced as practically racemic material as is generally the case with catalyst **3**. The pressure hydrogenation of ketones with two alkyl carbons adjacent to the carbonyl has never given good results using any catalyst, so substrate **11a** was attempted as a model for this, but no selectivity was observed. Reduction of α,α-dimethylpropiophenone **10a** gave alcohol product in slightly increased enantioselectivity (80% e.e. Table 2, Entry 2) relative to catalyst **3**. Reactions run at lower catalyst loading and shorter times reveal that, for this substrate, the activity is similar for both **3** and **8**. The reduced selectivity below S/C =200 is also a drawback sometimes observed with catalyst **3**; this behaviour will most likely preclude large scale applications in asymmetric catalysis until a solution is found. Ketone **12a**, deactivated by two bulky substituents and a heterocycle gives a chiral diol, with potential as a chiral ligand, or as a precursor to chiral ligands. Hydrogenation of **12a** with either catalyst **3** or **8** furnished the diol product in almost complete enantiopurity (the e.e. is amplified compared to that expected for the hydrogenation of a single carbonyl bond due the Horeau effect, whereby the formation of *meso* compound allows for the ‘removal’ of the undesired enantiomer [22]. This initial screen was sufficient to confirm that **8** is a competent alternative to catalyst **3**, although so far with no advantages, especially given its difficult synthesis.

**Table 3:** Enantioselective hydrogenation of ketone 13

**Entry**^**a**^	**Catalyst**	**Temp. (°C)**	**Conversion (%)**^**b**^	**e.e. (%)**^**c**^
1	**2**	70	21^d^	56
2	**3**	70	>99	49
3	**8**	70	37	50
4	**3**	50	14	54
5	**4**	70	<5	n.d.

^*a *^Reactions were carried out using 0.5 mol% preformed catalyst and 1 mol% KO^*t *^Bu at 70°C and 50 bar of hydrogen pressure with a 16 h reaction time unless indicated otherwise. ^*b *^Conversions were determined by ^1^H NMR analysis of the crude reaction mixtures (all peaks assigned). ^*c *^The e.e. value was determined by chiral HPLC (Chiralpak AD-H/Chiralcel OD-H column). The absolute configuration was determined to be *S *by comparison of optical rotation values with literature values. ^*d *^Base: catalyst of 50:1 used as is more common with Noyori catalysts.

**Scheme 5:**
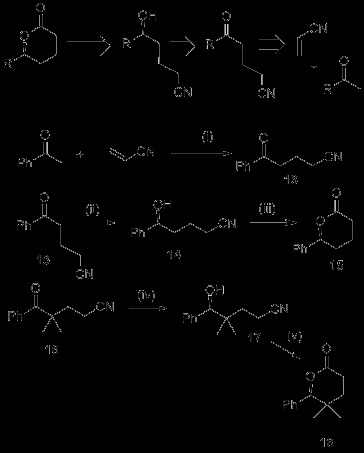
**A retrosynthesis of δ-lactones from commodity/bulk chemicals in 3 steps, and the reactions used to generate compound 15 and 18. **(i) 20 mol% cyclohexylamine, 7 mol% acetic acid, 0.2 mol% hydroquinone, 180°C, dropwise addition of acrylonitrile over 4 hours, heat for further 12 h (30% yield). (ii) Enantioselective hydrogenation according to Table 3. (iii) KOH (5 eq.), ethylene glycol, reflux, 3 days, then 10% HCl (62% yield). (iv) 0.5% catalyst **3**, 1% KOBu^t^. 50°C, 50 bar H_2_, IPA, 16 h, (>99% yield, 74% e.e.). (v) KOH (5 eq.), ethylene glycol, reflux, 24 h, then 10% HCl (76% yield, 74% e.e).

A particular interest in our research group is seeking to use catalytic methods to promote the synthesis of high-value-added functionalised molecules from bulk and commodity chemicals with maximum atom-efficiency. With this concept in mind, a rather narrow range of reaction types can be used to keep to these principles [23,24], but Michael addition and hydrogenation fit this criteria. A general synthesis of enantiomerically enriched delta-lactones might be possible from the bulk chemical acrylonitrile and methyl ketones as shown in Scheme 5. δ-lactones represent an important class of natural products with interesting applications in the flavour and fragrance industry in particular [25]. Their synthesis is generally quite labour intensive, although lactone **15** has previously been made by CBS reduction of a hydroxy-ester and ester hydrolysis-lactonisation [26]. Two of the key steps in our very concise synthesis represent quite underdeveloped chemistry. As far back as 1955, the organocatalytic Michael addition to acrylonitrile was reported in a patent [27], but no modern examples of organocatalytic acrylonitrile Michael additions exist. In our hands, this reaction only gave 30% yield of the desired ketone (and a brief screen of some other primary and secondary amines did not improve this). Whilst this substrate can be accessed by other routes in higher yield (see Additional file 1 and ref [28]), the organocatalytic Michael addition approach is worthy of a further research study given its atom-efficiency, its use of very simple, cheap reagents and the potential utility of the products. To the best of our knowledge, the asymmetric hydrogenation of nitrile-functionalised ketones does not appear in any of the plethora of publications on ketone hydrogenation, although there are reports of transfer hydrogenation of α-cyano-ketones [29]. There are probably two reasons for this gap in literature; Ru complexes would be envisaged to hydrogenate the nitrile [30], but also as we report in Scheme 5 and Table 3, this type of substrate seems to inhibit typical hydrogenation catalysts.

The asymmetric hydrogenation of the keto-nitrile **13** was studied using conditions that will readily reduce simple acetophenones. We found that the Noyori catalyst, **2** only gave a 21% conversion to product at 70°C with 56% e.e. (Table 3, Entry 1). On the other hand, catalyst **3** gave quantitative conversion to the desired alcohol, with complete chemoselectivity and a moderate 49% e.e. (Table 3, Entry 2). Lowering the temperature or switching to catalyst **8** resulted in lower conversion and no significant increases in enantioselectivity (Table 3, Entries 3–5). The *P,N,OH* catalyst, **4** was inactive. It is clear that the nitrile-ketones are extremely challenging substrates, although the St Andrews catalysts show distinct promise for this little studied but potentially useful class of ketone hydrogenations. We firmly encourage other researchers to improve on our efforts. The chiral alcohol produced was treated with KOH at high temperature resulting in formation of the lactone **15** in 62% yield. HPLC analysis and comparison of optical rotation data shows that this occurred with complete retention of stereochemistry. We briefly examined a gem-dimethyl analogue of **13** using catalyst **3** and were pleased to find it gave quantitative conversion and 74% e.e. for the product **17**, and that this could also be converted into lactone **18** with complete retention of stereochemistry.

## Conclusions

The important objective of comparing a different phosphine-amino-alcohol ligand to catalyst **4** has been accomplished, although this has ultimately delivered poor catalysts. The (*R,R*)-1,2-diphenylethylenediamine derived catalyst, while not offering large advantages over the previous systems developed in our laboratory is quite an effective catalyst that we now use in catalyst screening for more application-orientated studies. An example of a rather unique, but not yet viable, application of these catalysts is the delta lactone synthesis described; this potentially offers access to this class of molecules in three steps from the extremely cheap building blocks acrylonitrile and methyl-ketones. Both the organocatalytic acrylonitrile Michael addition and the asymmetric hydrogenation of nitrile-functionalised ketones are interesting unresolved target applications to spur the evaluation of new catalysts.

## Competing interests

The authors declare they have no competing interests.

## Authors’ information

Matt Clarke leads a research group that is developing greener scaleable catalytic synthetic methods at the University of St Andrews, EaStCHEM. The group works closely with industry. While the results of the groups work have been published in around 65 recent articles in the leading international chemistry journals, the authors are keen supporters of open-access journals that offer either free publication and access, or an institutional membership. Dr Scott Phillips completed his PhD in the Clarke group in 2011, specialising in asymmetric hydrogenation using Ru complexes of tridentate ligands. Dr José Fuentes completed his PhD in 2004 and is a post-doctoral research fellow and honorary lecturer in the Clarke group who is currently involved in several projects ranging from using supramolecular co-catalysts to optimise homogeneous catalytic reactions, the simultaneous control of regioselectivity and enantioselectivity in various alkene carbonylations and asymmetric hydrogenation reactions.

## Authors’ contributions

MC conceived of the projects described herein, assisted with experimental design and wrote the paper. SP carried out most of the ‘DPEN’ catalyst studies and the lactone synthesis. JF carried out the studies on the PNOH catalysts, prepared some substrates, contributed towards the synthetic studies on the DPEN catalyst and assisted in the writing of the paper. All authors read and approved the final manuscript.

## Supplementary Material

Addition file 1 **Full experimental details are available in Additional file **1**.** Click here for file
