# Supplementary material for: New phosphine-diamine and phosphine-amino-alcohol tridentate ligands for ruthenium catalysed enantioselective hydrogenation of ketones and a concise lactone synthesis enabled by asymmetric reduction of cyano-ketones
Source: Chem Cent J. 2012 Dec 10;6:151. doi: 10.1186/1752-153X-6-151 (PMC3538564; doi:10.1186/1752-153X-6-151)
Supplement: Addition file 1 — Full experimental details are available in Additional file 1. [file 1752-153X-6-151-S1.pdf]

Supporting Information

New phosphine-diamine and phosphine-amino-alcohol tridentate ligands for ruthenium catalysed enantioselective hydrogenation of ketones and a concise lactone synthesis enabled by an asymmetric reduction of cyano-ketones.

Jose A. Fuentes, Scott D. Phillips, and Matthew L. Clarke*

School of Chemistry, University of St Andrews, EaStCHEM, St Andrews, Fife, UK, KY16 9ST. Fax: +44 (0)1334 463808; Phone: +44 (0)1334 463850;

Experimental

Dry, degassed solvents were used for reactions that were carried out under an N_2 atmosphere unless otherwise indicated. Normal grade solvents were used for chromatography and work-up procedures under aerobic conditions. Solvents were removed by rotary evaporation on a Heidolph labrota 4000. Flash column chromatography (eluents given in brackets) was performed using Davisil silica gel Fluorochem 60 Å, particle size 35-70 micron. Thin-layer chromatography (TLC) was performed on pre-coated Aldrich TLC plates (POLYGRAM SIL G/UV_{254}). All microwave syntheses were carried out in a Biotage Initiator Microwave reactor using 5ml heavy-walled vials equipped with an air-tight septum. Melting points were determined with a Gallenkamp melting point apparatus Nº 889339 and are uncorrected. ^{1}H NMR, ^{13}C NMR and ^{31}P spectra were recorded either on a Bruker Avance 300 (^{1}H 300 MHz, ^{13}C 75.5 MHz and ^{31}P 121.4) or Bruker Avance 400 (^{1}H 400 MHz, ^{13}C 100 MHz and ^{31}P 162 MHz) instrument. ^{13}C NMR spectra were recorded using the DEPTQ sequence and internal deuterium lock. Chemical shifts are reported in ppm from tetramethyl silane (TMS) with the solvent resonance as the internal standard. Chemical shift values for ^{31}P spectra are reported downfield of phosphoric acid. Proton resonance multiplicities are given as s (singlet), d (doublet), t (triplet), q (quartet), m (multiplet), br (broad) or a combination of them. When appropriate, coupling constants (J) are quoted in Hz and are reported to the nearest 0.1Hz. All spectra were recorded at room temperature and the solvent for a particular spectrum is given in parentheses. Infrared spectra were recorded on a Perkin Elmer Paragon 1000 Spectrum GX FT-IR system. Compounds were analysed using disposable PTFE IR card with an aperture diameter of 15mm obtained from Aldrich. When disposable IR cards were not available, liquids were analysed as films, and solids were analysed as KBr disks. Absorptions maxima are reported in wavenumbers (cm^{-1}). Mass spectrometry was performed by the EPSRC National Mass Spectrometry Service Centre, Swansea University, using Waters ZQ4000, Thermofisher LTQ Orbitrap XL and Finnigan MAT 900 XLT instruments, or by Mrs Caroline Horsburgh at the University of St Andrews using a Waters Micromass GCT (Time of flight) fitted with lockspray for accurate mass (ESI) or GCT (CI). Only major peaks are reported and intensities are quoted as percentages of the base peaks. Optical rotations were measured on a Perkin Elmer 241 polarimeter using a 1ml cell with a 1 dm path length at room temperature using the sodium D-line, and a suitable solvent that is reported along with the concentration (c = g/100ml). Microanalysis for carbon, hydrogen and nitrogen were performed using a EA 1110 CHNS CE instruments elemental analyser by Mrs Sylvia Williamson or Miss Donna McColl at the University of St Andrews or by Mr Stephen Boyer at the London Metropolitan University. HPLC analysis has been determined using a Varian

Prostar operated by Galaxie workstation PC software. Catalysts **3** and **4** were prepared according to our published procedures[2a, b]

General Reagents

Dichlorotetrakis(dimethyl sulfoxide) ruthenium (II)

Prepared by modification of a literature procedure.[1] Ruthenium trichloride trihydrate (0.86 g, 4.13 mmol) was refluxed in dimethyl sulfoxide (5 mL) for 5 min under nitrogen. The volume was then reduced to half *in vacuo* and acetone added prompting formation of a yellow solid. The precipitate was filtered off, washed with acetone and dried *in vacuo*. Recrystallisation from dimethyl sulfoxide yielded complex (0.87 g, 44 %) as yellow crystals, mp 190 °C (lit.[1] 193 °C).

General procedure for synthesis of the tridentate PNO ligands

To a solution of the corresponding 1-amino-2-indanol (0.298 g, 2.00 mmol) in ethanol (6 ml), a solution of 2-(diphenylphosphino)benzaldehyde (0.290 g, 1.00 mmol) in ethanol (6 mL) was added under a N_2 atmosphere. The resulting yellow solution was stirred at room temperature for 3 hours and then sodium borohydride (0.302g, 8 mmol) was added. The reaction mixture was stirred for a further 16 hours and then the solvent was removed under vacuum. The resulting residue was dissolved in dichloromethane (20 mL) and quenched with a saturated solution of ammonium chloride (10 mL). After extraction of the aqueous phase with dichloromethane (1 x 10 mL) under a N_2 atmosphere, the combined organic phases were dried using magnesium sulphate anhydrous and the solvent was then removed under vacuunm to afford the desired product as a pale yellow solid. The ligand can also be further purified by chromatography on silica gel using a mixture of diethy ether and hexane 4:1 as eluent.

(1*R*, 2*S*)-PNO Ligand

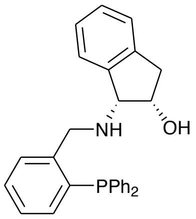

Pale yellow solid. (423 mg, 89%). Mp 47-50 °C; $[\alpha]_D^{20}$ –2.1 (*c* 0.39, $CHCl_3$); IR ($CDCl_3$, cm^{-1}) 3404, 3057, 2910, 1642, 1586, 1478, 1434, 1385, 1309, 1264, 1176, 1119, 1089 cm^{-1}; ^{1}H NMR (300 MHz, $CDCl_3$) δ= 2.82 (1H, dd, *J* 2.7, 16.4 Hz, CH_2), 2.91 (1H, dd, *J* 5.0, 16.4 Hz, CH_2), 3.98-4.00 (1H, m, CH-NH), 4.07 (1H, d, *J* 12.8 CH_2-NH), 4.13 (1H, dd, *J* 1.8, 12.8 CH_2-NH), 4.32-4.36 (1H, m, CH-OH), 6.79 (1H, d, *J* 7.5 NH), 6.87 (1H, ddd, *J* 1.2, 4.5, 7.7 ArCH), 7.10-7.44 (17H, m, ArCH); ^{13}C NMR (75 MHz, $CDCl_3$) δ= 40.0 (CH_2), 51.8 (d, $^3J_{C-P}$ 20.0 Hz, CH_2), 65.7 (CH), 71.2 (CH), 124.2 (ArCH), 125.9 (ArCH), 127.0 (ArCH), 128.3 (ArCH), 129.1 (d, *J* 4.5 Hz, ArCH), 129.2 (d, *J* 4.5 Hz, ArCH), 129.3 (d, *J* 4.0 Hz, ArCH), 129.7 (ArCH), 130.3 (d, *J* 5.0 Hz, ArCH), 134.2 (d, *J* 3.0 Hz, ArCH), 134.4 (d, *J* 3.0 Hz, ArCH), 134.5 (ArCH), 136.3-137.0 (m, 3xArC), 141.5 (ArC), 142.6 (ArC), 144.2 (d, *J* 24.4 Hz, ArC); ^{31}P{^{1}H} NMR (121 MHz, $CDCl_3$) δ= –16.32 (s); MS (TOF ESI) m/z: 446.0 ($[MNa]^+$, 41%), 423($[MH]^+$, 100), 275.0 (8); Found (TOF ESI) 424.1842 ($[MH]^+$), $C_{28}H_{27}NOP$ requires 424.1830.

(1*S*, 2*R*)-PNO Ligand

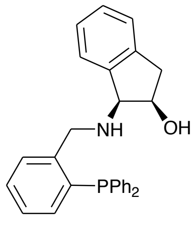

Pale yellow solid (206 mg, 97%). Mp 48-50 ºC; $[\alpha]_D^{20}$ +2.2 (*c* 0.275, $CHCl_3$); IR ($CDCl_3$, cm^{-1}) 3343, 3057, 2912, 1586, 1477, 1435, 1089 cm^{-1}; ^1H NMR (300 MHz, $CDCl_3$) δ= 2.81 (1H, dd, *J* 2.7, 16.4 Hz, CH_2), 2.91 (1H, dd, *J* 5.0, 16.4 Hz, CH_2), 3.97-3.99 (1H, m, CH-NH), 4.06 (1H, d, *J* 12.9 CH_2-NH), 4.12 (1H, dd, *J* 1.8, 13.0 CH_2-NH), 4.31-4.36 (1H, m, CH-OH), 6.78 (1H, d, *J* 7.5 NH), 6.87 (1H, ddd, *J* 1.2, 4.5, 7.6 ArCH), 7.10-7.44 (17H, m, ArCH); ^{13}C NMR (75 MHz, $CDCl_3$) δ= 40.0 (CH_2), 51.8 (d, $^3J_{C-P}$ 20.0 Hz, CH_2), 65.7 (CH), 71.3 (CH), 124.2 (ArCH), 125.9 (ArCH), 127.0 (ArCH), 128.3 (ArCH), 129.1 (d, *J* 4.4 Hz, ArCH), 129.2 (d, *J* 4.4 Hz, ArCH), 129.4 (d, *J* 4.0 Hz, ArCH), 129.8 (ArCH), 130.2 (d, *J* 5.4 Hz, ArCH), 134.2 (d, *J* 2.6 Hz, ArCH), 134.4 (d, *J* 2.7 Hz, ArCH), 134.5 (ArCH), 136.3-137.0 (m, 3xArC), 141.5 (ArC), 142.6 (ArC), 144.3 (d, *J* 24.3 Hz, ArC); $^{31}P\{^1H\}$ NMR (121 MHz, $CDCl_3$) δ= –16.27 (s); MS (TOF CI) m/z: 424.2 ($[MH]^+$, 100%), 290.1 (10), 286.0 (19), 276.1 (10); Found (TOF CI) 424.1819 ($[MH]^+$), $C_{28}H_{27}NOP$ requires 424.1830.

(1*S*, 2*S*)-PNO Ligand

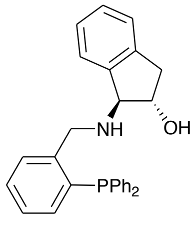

Pale yellow solid. (95 mg, 75%). Mp 53-54 ºC; $[\alpha]_D^{20}$ +7.7 (*c* 0.3, $CHCl_3$); IR ($CDCl_3$, cm^{-1}) 3375, 3020, 2912, 1656, 1523, 1435 cm^{-1}; ^1H NMR (400 MHz, $CDCl_3$) δ= 2.11 (1H, br s, OH), 2.67 (1H, dd, *J* 6.2, 15.8 Hz, CH_2), 3.11 (1H, dd, *J* 6.8, 15.8 Hz, CH_2), 3.93-3.94 (1H, m, CH-NH), 4.05 (1H, d, *J* 12.9 CH_2-NH), 4.17-4.23 (2H, m, CH_2-NH, CH-OH), 6.84 (1H, ddd, *J* 1.0, 4.6, 7.6 ArCH), 7.10-7.44 (18H, m, ArCH, NH); ^{13}C NMR (101 MHz, $CDCl_3$) δ= 38.8 (CH_2), 50.2 (d, $^3J_{C-P}$ 19.9 Hz, CH_2), 70.1 (CH), 78.9 (CH), 124.4 (ArCH), 125.0 (ArCH), 126.8 (ArCH), 127.7 (d, *J* 16.7 Hz, ArCH), 128.6 (d, *J* 4.3 Hz, ArCH), 128.7 (d, *J* 4.4 Hz, ArCH), 128.9 (d, *J* 6.8 Hz, ArCH), 129.6 (ArCH), 133.7-134.1 (m, 3xArCH), 137.8 (d, *J* 12.5 Hz, ArC), 136.6-136.7 (m, 2xArC), 140.0 (ArC), 142.5 (ArC), 144.6 (d, *J* 23.7 Hz, ArC); $^{31}P\{^1H\}$ NMR (162 MHz, $CDCl_3$) δ= –15.61 (s); MS (TOF CI) m/z: 424.2 ($[MH]^+$, 100%), 406.2 (11), 309.1 (5), 275.1 (5); Found (TOF CI) 424.1899 ($[MH]^+$), $C_{28}H_{27}NOP$ requires 424.1830.

General procedure for synthesis of PNO Ru complexes

$[RuCl_2(DMSO)_4]$ (183 mg, 0.38 mmol) was placed in a microwave tube under a N_2 atmosphere. The coorresponding PNO ligand (0.38 mmol) was then added *via* syringe as a solution in THF (5 mL). The reaction mixture was then heated using microwave radiation at 120 ˚C for 15 min. The solvent from the resulting orange solution was then partially removed under vacuum to a volume of approximately 1 mL and then hexane (3 mL) was added to obtain the desired complex as an orange-brown precipitate. The

complex can also be further purified by chromatography on silica gel using a mixture of dichloromethane and acetone 1:1 as eluent.

(1*R*, 2*S*)-5

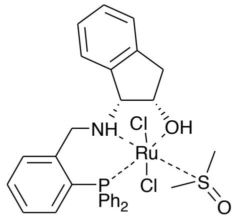

Orange solid (178 mg, 70%). Mp >195 ºC; $[\alpha]_D^{20}$ +140 (*c* 0.25, $CHCl_3$); IR ($CDCl_3$, cm^{-1}) 3211, 3062, 2915, 1650, 1484, 1434 cm^{-1}; ^1H NMR (300 MHz, $CDCl_3$) δ= 2.74 (3H, s, CH_3), 2.04 (1H, dd, *J* 7.5, 15.0 Hz, CH_2), 3.49 (1H, dd, *J* 9.0, 15.0 Hz, CH_2), 3.15 (3H, s, CH_3), 4.04-4.57 (3H, m, CH_2-NH), 4.80-4.85 (1H, m, CH), 4.96 (1H, m, CH), 5.70 (1H, br s, OH), 6.88-6.94 (1H, m, ArCH), 7.12-7.44 (17H, m, ArCH); ^{13}C NMR (75 MHz, $CDCl_3$) δ= 35.8 (CH_2), 44.5 (CH_3), 45.1 (CH_3), 53.1 (d, $^3J_{C-P}$ 6.5 Hz, CH_2), 67.0 (CH), 77.8 (CH), 124.7 (ArCH), 125.2 (ArCH), 126.3-133.9 (16xArCH), 128.4 (d, *J* 48.9 Hz, ArC), 131.1 (d, *J* 46.2 Hz, ArC), 133.3 (d, *J* 50.0 Hz, ArC), 137.2 (ArC), 139.1 (d, *J* 15.8 Hz, ArCH), 139.3 (ArC); $^{31}P\{^1H\}$ NMR (121 MHz, $CDCl_3$) δ= +57.9 (s); MS (TOF ES) m/z: 671.8 ([M-H]$^-$, 100%); Found (TOF ES) 672.0239 ([M-H]$^-$), $C_{30}H_{31}NO_2PSCl_2Ru$ requires 672.0234.

(1*S*, 2*R*)-5

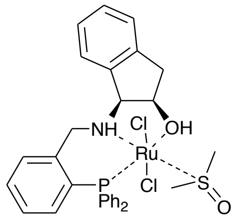

Orange solid (85 mg, 67%). Mp >195 ºC; $[\alpha]_D^{20}$ −134 (*c* 0.275, $CHCl_3$); IR ($CDCl_3$, cm^{-1}) 3211, 3062, 2922, 1617, 1571, 1485, 1475, 1436, 1311, 1216, 1093 cm^{-1}; ^1H NMR (300 MHz, $CDCl_3$) δ= 2.71 (3H, s, CH_3), 3.05-3.12 (1H, m, CH_2), 3.13 (3H, s, CH_3), 3.51 (1H, dd, *J* 8.6, 15.1 Hz, CH_2), 4.04-4.58 (3H, m, CH_2-NH), 4.81-4.87 (1H, m, CH), 5.00 (1H, m, CH), 5.38 (1H, br s, OH), 6.91-6.97 (1H, m, ArCH), 7.11-7.48 (17H, m, ArCH); ^{13}C NMR (100 MHz, $CDCl_3$) δ= 36.8 (CH_2), 45.6 (CH_3), 46.2 (CH_3), 54.2 (d, $^3J_{C-P}$ 6.5 Hz, CH_2), 68.1 (CH), 78.9 (CH), 125.7 (ArCH), 126.2 (ArCH), 127.3-134.9 (16xArCH), 129.4 (d, *J* 49.4 Hz, ArC), 132.1 (d, *J* 45.9 Hz, ArC), 134.3 (d, *J* 49.9 Hz, ArC), 138.3 (ArC), 140.1 (d, *J* 15.4 Hz, ArCH), 140.3 (ArC); $^{31}P\{^1H\}$ NMR (161 MHz, $CDCl_3$) δ= +58.0 (s); MS (NSI) m/z: 674.0 ([MH]$^+$, 100%), 560.0 (9), 440.2 (45), 279.2 (21), 217.2 (12); Found (NSI) 668.0423 ([MH]$^+$), $C_{30}H_{33}NO_2PSCl_2Ru$ requires 668.0417; Found: C, 53.56; H, 4.72, N, 1.96. Calc. for $C_{30}H_{32}Cl_2NO_2PSRu$: C, 53.49; H, 4.79, N, 2.08.

(1*S*, 2*S*)-6

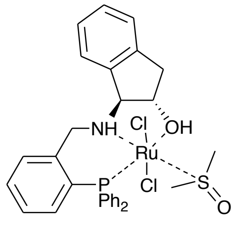

Orange solid (50 mg, 78%). Mp >195 ºC; $[\alpha]_D^{20}$ –206.7 (*c* 0.3, $CHCl_3$); IR ($CDCl_3$, cm^{-1}) 3421, 3062, 2965, 1636, 1484, 1461, 1435, 1310, 1206 cm^{-1}; ^1H NMR (400 MHz, $CDCl_3$) δ= 2.58 (3H, s, CH_3), 2.83-2.91 (1H, m, CH_2), 3.00-3.05 (1H, m, CH_2), 3.23 (3H, s, CH_3), 4.23-4.31 (1H, m, NH), 4.56-4.67 (3H, m, CH_2, OH), 4.86-4.93 (1H, m, CH), 5.05-5.12 (1H, m, CH), 7.04-7.11 (1H, m, ArCH), 7.13-7.67 (17H, m, ArCH); ^{13}C NMR (100 MHz, $CDCl_3$) δ= 34.6 (CH_2), 45.7 (CH_3), 46.8 (CH_3), 55.4 (d, $^3J_{C-P}$ 6.4 Hz, CH_2), 69.2 (CH), 82.5 (CH), 122.3 (ArCH), 126.4 (ArCH), 127.4-134.9 (16xArCH), 130.0 (d, *J* 51.9 Hz, ArC), 130.9 (d, *J* 44.8 Hz, ArC), 133.8 (d, *J* 51.8 Hz, ArC), 138.1 (ArC), 140.1(ArC), 141.0 (d, *J* 14.1 Hz, ArC); $^{31}P\{^1H\}$ NMR (162 MHz, $CDCl_3$) δ= 58.68 (s); MS (NSI) m/z: 674.0 ($[MH]^+$, 100%), 440.2 (76), 279.2 (16), 217.2 (12) ; Found (NSI) 668.0417 ($[MH]^+$), $C_{30}H_{33}NO_2PSCl_2Ru$ requires 668.0417; Found: C, 53.41; H, 4.70, N, 2.02. Calc. for $C_{30}H_{32}Cl_2NO_2PSRu$: C, 53.49; H, 4.79, N, 2.08.

Complex (*R*,*R*)-8

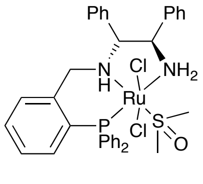

The DPEN-derived ligand (*R*,*R*)-**7** was prepared in the same manner as the ligand precursor of complex **3**.[2] NMR analysis (EtOH, C_6D_6) of the reaction mixture before treatment with sodium borohydride showed two species at -8.1 (Imine) and -15.8 ppm in a 1.3:1 ratio. After treatment with sodium borohydride for 16h and work-up, the ^{31}P NMR spectrum showed the presence of several species at δ_P (DCM, C_6D_6) = -9.7, -15.8 and -16.2 ppm. A purification attempt was performed using silica gel and a solvent gradient starting from Hexane\EtOAc 1:1, then Hexane\EtOAc 1:3, and finally EtOAc/MeOH 4:1. A fraction (δ_P ($CDCl_3$) = -16.0) was obtained (80 mg, 14%) and it was decided to use it directly in the complexation reaction. A solution of the ligand in THF (5 mL) was added to a microwave vial containing 1 eq of $[RuCl_2(DMSO)_4]$ and heated to 110°C for 1h. The reaction gave one main phosphorus containing species at δ_P (THF, C_6D_6) = +46.4. Column chromatography on silica (DCM:acetonitrile 4:1) gave analytically pure complex (*R*,*R*)-**8** as an orange solid (45 mg, 38 %), m.p. 187-189°C (decomp.). Found: C, 56.86; H, 4.82; N, 3.68%; $C_{35}H_{37}Cl_2N_2OPRuS$ requires C, 57.06; H, 5.06; N, 3.80%; $[\alpha]_D^{20}$ +79.3 (*c* 0.2, $CHCl_3$); ν_{max}/cm^{-1} (IR card) 3411 (br, s), 1278 (br, s), 1179 (s), 1061 (m), 700 (m), 573 (w), 534 (w), 432 (w) and 415 (w); δ_H (400 MHz, $CDCl_3$) † 7.59-7.41 (4H, m, $C_{Ar}H$), 7.37-7.22 (10H, m, $C_{Ar}H$), 7.18-7.03 (8H, m, $C_{Ar}H$), 6.77-6.67 (1H, m, $C_{Ar}H$), 4.78-4.70 (1H, m, C*H*N), 4.51 (1H, br s, N*H*), 4.43-4.27 (3H, m, CH_AH_BAr +NH_2), 4.13-4.06 (1H, m, C*H*N), and 3.54-3.44 (1H, m, CH_AH_BAr); δ_C (100 MHz, $CDCl_3$)‡ 140.6 (d, *J* 16, C_{ipso}), 139.4 (C_{ipso}), 136.7 (C_{ipso}), 135.6 (C_{ipso}), 135.3 ($C_{Ar}H$), 135.2 ($C_{Ar}H$), 134.0 ($C_{Ar}H$), 132.9 (C_{ipso}), 132.7 ($C_{Ar}H$), 132.5 (C_{ipso}), 130.8 ($C_{Ar}H$), 130.7 ($C_{Ar}H$), 130.0 ($C_{Ar}H$), 130.0 ($C_{Ar}H$), 128.9 ($C_{Ar}H$), 128.6 ($C_{Ar}H$), 128.3 ($C_{Ar}H$), 128.2 ($C_{Ar}H$), 128.2 ($C_{Ar}H$), 127.8 ($C_{Ar}H$), 127.6 ($C_{Ar}H$), 127.4 ($C_{Ar}H$), 71.6 (*C*HN), 63.2 (*C*HN), 54.1 (d, *J* 7, CH_2Ar), 47.0 ($C(H_A)_3SOC(H_B)_3$) and 45.3 ($C(H_A)_3SOC(H_B)_3$); δ_P (162 MHz, $CDCl_3$) +42.4; *m/z* (ES+) 623.1 ($[M\text{-}Cl\text{-}DMSO]^+$, 100%); HRMS (ES+) found 623.0956, $[C_{33}H_{31}ClN_2PRu]^+$ requires 623.0956. †Assignments supported by ^1H-^1H COSY, ^1H-^{13}C HSQC and ^1H-^{13}C HMBC correlations. ‡Assignments supported by ^1H-^{13}C HSQC and HMBC correlations.

Hydrogenation Using $[RuCl_2(P\text{^}N\text{^}X)L]$ Catalysts

A solution of substrate (*ca* 1 mmol), catalyst and potassium *tert*-butoxide (1 M solution in 2-methyl-2-propanol) in degassed isopropanol (3 mL) was prepared in a microwave vial under an atmosphere of nitrogen. The microwave tube was then placed inside a steel autoclave with two syringe needles piercing the lid of the vial. The autoclave was then sealed and flushed

three times with hydrogen before being charged with hydrogen to the required pressure. The reactions were stirred at the same speed for the desired times at the required temperature using a stainless steel heating jacket connected to a thermocouple and heater. After the desired time passed, the autoclave was opened and the reaction mixture concentrated *in vacuo*. The conversion of substrate to product was calculated by ^{1}H NMR spectroscopy (In these experiments only starting material and product were observed, so an internal standard was generally not used). The products were isolated by column chromatography, or simple filtration through a 3 cm pad of silica with ether eluent, or short-path distillation and characterised by comparison of NMR, IR, MS, optical rotation and where appropriate melting point data, with authentic samples. The enantiopurity of the product (where applicable) was determined using high performance liquid chromatography with the chiral stationary phase noted for each product.

Racemic Reduction with Sodium Borohydride

All alcohols produced were also reduced with sodium borohydride on a small scale to develop the HPLC method. To a solution of the substrate (1 equivalent) in absolute ethanol was added powdered sodium borohydride (3 equivalents) in small portions to avoid vigorous reaction and the mixture stirred. The reaction was monitored by thin layer chromatography and upon completion quenched with 10 % hydrochloric acid solution. The mixture was then extracted with dichloromethane and washed with water and brine before drying with magnesium sulfate, filtration and concentration *in vacuo* yielded the product. The TLC, HPLC and NMR data is in agreement with the products from the hydrogenation reactions.

1-Phenylethanol (9b)

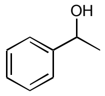

$[\alpha]_D^{20}$ -32.0 (60% e.e., *c* 2.5, $CHCl_3$) (lit.[7] $[\alpha]_D^{25}$ -45.0 (*S*, 99% e.e., *c* 5.0, methanol)); δ_H (400 MHz, $CDCl_3$) 7.18-7.33 (5H, m, $C_{Ar}H$), 4.84 (1H, q, *J* 6, C*H*OH), 1.63 (1H, br s, -O*H*) and 1.43 (3H, d, *J* 6, CH_3). ^{13}C NMR (75 MHz, $CDCl_3$) δ= 25.19 (CH_3), 70.46 (*C*HOH), 125.41 (Ar *C*), 127.51 (Ar *C*), 128.54 (Ar *C*), 145.83 (Ar *C*). Enantioselectivity determined by HPLC, ChiralPak OD-H, 0.5 mL/min, 95:5 hexane:2-propanol. Retention times: 17.4 min ((*R*)-(+)-enantiomer) and 21.2 min ((*S*)-(–)-enantiomer). Data are in agreement with the literature.[8]

2,2-Dimethyl-1-phenylpropan-1-ol (10b)

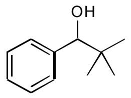

m.p. 45 °C (lit.[9] 45 °C); $[\alpha]_D^{20}$ -19.3 (74% e.e., *c* 0.3, acetone) (lit.[10] $[\alpha]_D^{25}$ -30.3 (*S*, >99% e.e., *c* 0.36, acetone); δ_H (300 MHz, $CDCl_3$) 7.25-7.19 (5H, m, $C_{Ar}H$), 4.31 (s, 1H, C*H*OH), 1.97 (1H, br s, CHO*H*) and 0.85 (9H, s, $C(CH_3)_3$); ^{13}C NMR (100 MHz, $CDCl_3$) δ= 25.93 (CH_3), 35.64 (CMe_3), 82.42 (*C*HOH), 127.30 (Ar *C*), 127.57 (Ar *C*), 127.61 (Ar *C*), 142.20 (Ar *C*). Enantioselectivity determined by chiral HPLC. Chiralpak OD-H, 1 mL/min, 98:2 hexane:2-propanol. Retention times: 10.5 min (*S*, major enantiomer) and 15.0 (*R*, minor enantiomer). Data are in agreement with the literature.[8]

1,1'-(furan-2,5-diyl)bis(2,2-dimethylpropan-1-one) (12a)

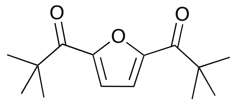

Furan (20 mmol, 1.36g) was placed in a two necked flask under a nitrogen atmosphere. Hexane (10 mL) and BuLi (62.5 mmol, 39 mL, 1.6 M solution in hexane) were added and the reaction mixture heated at 70 °C in an oil bath for 1.5 h. After that time, the flask was taken out of the oil bath and pivalonitrile (50 mmol, 4.15 g) was added. The reaction mixture was stirred for 1.5 h and the quenched with HCl 1M (20 ml). After extraction of the aqueous phase with diethtl ether (3 x 30 mL), the combined organic phases were dried using magnesium sulphate anhydrous and the solvent was then removed under vacuunm. The ketone was purified by chromatography on silica gel using a mixture of diethy ether and hexane 1:5 as eluent to give the product as a pale yellow solid (0.78 g, 3.3 mmol, 16%). ^{1}H NMR (400 MHz, $CDCl_3$) δ= 1.40 (18H, s, 6xCH_3), 7.23 (2H, s, 2xArCH); ^{13}C NMR (100 MHz, $CDCl_3$) δ= 26.6 (6xCH_3), 43.2 (2xC(CH_3)), 118.5 (2xArCH), 152.8 (2xArC), 195.1 (2xC=O); MS (TOF ES) m/z: 259.0 ([MNa]$^+$. Data are in agreement with the literature.[3]

1,1'-(Furan-2,5-diyl)bis(2,2-dimethylpropan-1-ol) (12b)

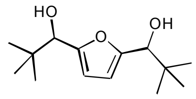

Mp 67-68 ºC; IR (KBr, cm^{-1}) 3313, 2953, 2868, 1560, 1479, 1462, 1392, 1364, 1300, 1236, 1199, 1050, 1009, 968, 897 cm^{-1}; ^{1}H NMR (400 MHz, $CDCl_3$) δ= 0.94 (18H, s, 6xCH_3), 2.10 (2H, s, 2xOH), 4.32 (2H, s, 2xCH), 6.15 (2H, s, 2xArCH); ^{13}C NMR (100 MHz, $CDCl_3$) δ= 25.8 (6xCH_3), 35.7 (2xC(CH_3)), 76.4 (2xCH), 107.5 (2xArCH), 154.7 (2xArC); MS (TOF ES) m/z: 262.9 ([MNa]$^+$; Found (TOF ES) 263.1621 ([MNa]$^+$), $C_{14}H_{24}O_3Na$ requires 263.1623. Meso: ^{1}H NMR (400 MHz, $CDCl_3$) δ= 0.94 (18H, s, 6xCH_3), 2.30 (2H, s, 2xOH), 4.29 (2H, s, 2xCH), 6.13 (2H, s, 2xArCH); ^{13}C NMR (100 MHz, $CDCl_3$) δ= 25.9 (6xCH_3), 35.7 (2xC(CH_3)), 76.5 (2xCH), 107.56 (2xArCH), 154.5 (2xArC); Enantioselectivity determined by chiral HPLC. ChiralPak AD, 1.0 miL/min, 93:7 hexane:2-propanol. Retention times 10.4 min (major enantiomer), 14.8 min (meso), 21.4 min (minor enantiomer).

Ethyl 2-cyano-5-oxo-5-phenylpentanoate

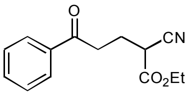

Prepared following the procedure of Wessig.[4] To a solution of 1-phenylprop-2-ene-1-one (0.841 g, 6.40 mmol) and ethyl cyanoacetate (0.677 mL, 6.40 mmol) in tetrahydrofuran wass added potassium carbonate (0.089 g, 6.50 mmol) and 18-crown-6 (0.142 mL, 0.66 mmol) and the mixture stirred for 2 h. After this period, water was added and the mixture diluted with diethyl ether. The organic component was separated, washed with further water, dried over magnesium sulfate, filtered, and concentrated *in vacuo* giving product that was deemed pure enough for direct use in the next step. δ_H (400 MHz, $CDCl_3$) 8.00-7.94 (2H, m, $C_{Ar}H$), 7.62-7.56 (1H, m, $C_{Ar}H$), 7.50-7.44 (2H, m, $C_{Ar}H$), 4.26 (2H, q, *J* 7, CH_2CH_3), 3.82 (1H, dd, *J* 10, 7, C*H*), 3.25 (2H, t, *J* 7, $COCH_2$), 2.56-2.24 (2H, m, $COCH_2CH_2$) and 1.32 (3H, t, CH_2CH_3). Data are in agreement with the literature.[4]

5-Oxo-5-phenylpentanenitrile (13)

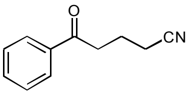

Following the procedure of Wessig,[4] ethyl 2-cyano-5-oxo-5-phenylpentanoate (0.90 g, 3.7 mmol), sodium chloride (0.20 g, 3.8 mmol) and water (2 mL) in dimethylsulfoxide (20 mL) were refluxed for 24 h. After this time, the mixture was diluted with further water and extracted with diethyl ether. The organic component was dried over magnesium sulfate, filtered and the solvent removed *in vacuo*. Flash chromatography on silica (10:2 petroleum ether:ethyl acetate) furnished the product as a colourless oil (0.320 g, 50%); δ_H (300 MHz, $CDCl_3$) 7.99 (2H, d, *J* 7, $C_{Ar}H$), 7.66-7.55 (1H, m, $C_{Ar}H$), 7.55-7.44 (2H, m, $C_{Ar}H$), 3.20 (2H, t, *J* 7, CH_2), 2.54 (2H, t, *J* 7, CH_2) and 2.13 (2H, app pent, *J* 7, CH_2); δ_C (75 MHz, $CDCl_3$) 198.3 (*C*=O), 136.5 (C_{ipso}), 133.6 (C_{Ar}H), 128.8 (C_{Ar}H), 128.1 (C_{Ar}H), 119.6 (*C*N), 36.5 (CH_2), 19.9 (CH_2) and 16.6 (CH_2). *m/z* (ES+) 196.07 ($(M+Na)^+$, 100%); HRMS (ES+) found 196.0743, $[C_{11}H_{11}NONa]^+$ requires 196.0738. Data are in agreement with the literature.[4]

5-Hydroxy-5-phenylpentanenitrile (14)

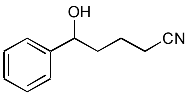

$[\alpha]_D^{20}$ -20.4 (49% ee, *c* 4.5, $CHCl_3$); δ_H ($CDCl_3$, 300 MHz) 7.40-7.18 (5H, m, $C_{Ar}H$), 4.69-4.63 (1H, s, C*H*OH), 2.34-2.28 (2H, m, CH_2CN), 2.00 (1H, br s, CHO*H*) and 1.90-1.67 (4H, m, CH_2CH_2); δ_C ($CDCl_3$, 75 MHz) 144.0 (C_{ipso}), 128.8 (C_{Ar}H), 128.0 (C_{Ar}H), 125.8 (C_{Ar}H), 119.7 (*C*N), 73.7 (*C*HOH), 37.7 (CH_2), 21.9 (CH_2) and 17.2 (CH_2); *m/z* (ES+) 198.09 ($[M+Na]^+$, 100 %); HRMS (ES+) 198.0894 ($[M+Na]^+$), $[C_{11}H_{13}NONa]^+$ requires 198.0895. Enantioselectivity determined by chiral HPLC. Chiralcel OD-H, 1 mL/min, 90:10 hexane:2-propanol. Retention times 20.2 min (major enantiomer), 22.1 min (minor enantiomer). Data are in agreement with the literature for the racemic compound that has been reported before.[11]

6-Phenyltetrahydro-2*H*-pyran-2-one (15)

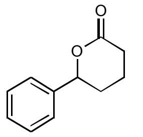

$[\alpha]_D^{20}$ -16.6 (48 % e.e., *c* 0.1, $CHCl_3$) (lit.[12] $[\alpha]_D^{25}$ +38.5 (*R,* 98 % e.e., *c* 1.0, $CHCl_3$); δ_H ($CDCl_3$, 400 MHz) 7.45-7.24 (5H, m, $C_{Ar}H$), 5.30 (1H, dd, *J* 11,3, C*H*O), 2.80-2.46 (2H, m, CH_2), 2.20-2.10 (1H, m, CH_AH_B), 2.00-1.81 (3H, m, CH_AH_B + CH_2); Enantioselectivity determined by chiral HPLC. Chiralcel OD-H, 1 mL/min, 90:10 hexane:2-propanol. Retention times 28.7 min (minor enantiomer) and 32.8 min (major enantiomer). Data are in agreement with the literature.[13]

4,4-Dimethyl-5-oxo-5-phenylpentanenitrile (16)

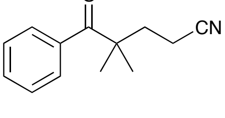

In a similar procedure to that reported by Campbell,[5] acrylonitrile (2.62 mL, 0.04 mol) was added dropwise with stirring to a solution of isobutyrophenone (6.00 mL, 0.04 mol) in dioxane (10 mL) containing 30 % solution of potassium hydroxide in methanol (0.5 mL). This solution was heated at 50 °C for 24 h. After this time, the mixture was poured into water and the oil separated. Column chromatography of this oil on silica (Hexane:EtOAc 9:1) yielded the title compound as a colourless oil (1.71 g, 21 %). δ_H ($CDCl_3$, 300 MHz) 7.72-7.65 (2H, m, $C_{Ar}H$), 7.55-7.40 (3H, m, $C_{Ar}H$), 2.39-2.30 (2H, m, CH_2), 2.18-2.10, (2H, m, CH_2) and 1.42 (6H, s, $C(CH_3)_2$; δ_C ($CDCl_3$, 75 MHz) 207.0 (*C*=O), 137.9 (C_{ipso}), 131.6 ($C_{Ar}H$), 128.4 ($C_{Ar}H$), 127.8 ($C_{Ar}H$), 119.8 (*C*≡N), 47.0 ($C(CH_3)_2$), 36.4 (CH_2), 25.8 ($C(CH_3)_2$) and 13.1 (CH_2); *m/z* (ES+) 224.01 ($[M+Na]^+$, 100 %) and 425.03 ($[2M+Na]^+$, 25). Data are in agreement with the literature.[6]

5-Hydroxy-4,4-dimethyl-5-phenylpentanenitrile (17)

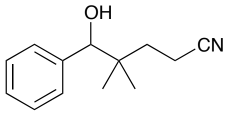

$[\alpha]_D^{20}$ -3.6 (74% ee, *c* 1.7, $CHCl_3$); δ_H ($CDCl_3$, 400 MHz) 7.32-7.17 (5H, m, $C_{Ar}H$), 4.35 (1H, s, *CH*OH), 2.33 (1H, app dd, *J* 7, 2, $C(H_A)(H_B)CN$), 2.31 (1H, app dd, *J* 7, 1, $C(H_A)(H_B)CN$), 1.92 (1H, br s, CHO*H*), 1.84-1.75 (1H, m, $C(H_A)(H_B)CH_2CN$), 1.64-1.54 (1H, m, $C(H_A)(H_B)CH_2CN$), 0.82 (3H, s, $C(C_AH_3)(C_BH_3)$) and 0.81 (3H, s, $C(C_AH_3)(C_BH_3)$); δ_C ($CDCl_3$, 101 MHz) 141.3 (C_{ipso}), 127.9 ($C_{Ar}H$), 127.8 ($C_{Ar}H$), 127.6 ($C_{Ar}H$), 120.8 (*C*≡N), 80.9 (*C*HOH), 37.9 ($C(CH_3)_2$), 34.4 (CH_2), 23.4 $C(C_AH_3)(C_BH_3)$, 22.3 $C(C_AH_3)(C_BH_3)$ and 12.6 (CH_2); *m/z* (ES+) 226.12 ($[M+Na]^+$, 100 %). Enantioselectivity determined by chiral HPLC. Chiralpak AD, 1 mL/min, 90:10 hexane:2-propanol. Retention times 12.2 min (minor enantiomer), 15.2 min (major enantiomer). Data are in agreement with the literature for the racemic compound that has been prepared previously.[14]

5,5-Dimethyl-6-phenyltetrahydro-2*H*-pyran-2-one (18)

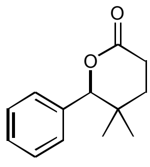

m.p. 103-104 °C (lit.[15] 102-103 °C); $[\alpha]_D^{20}$ -17.2 (74% ee, *c* 0.5, $CHCl_3$); δ_H ($CDCl_3$, 400 MHz) 7.29-7.22 (2H, m, $C_{Ar}H$), 7.21-7.14 (3H, m, $C_{Ar}H$), 5.00 (1H, s, >C*H*O-), 2.65-2.60 (2H, m, CH_2CO), 1.86-1.75 (1H, m, $C(H_A)(H_B)CH_2CO$), 1.70 (1H, m, $C(H_A)(H_B)CH_2CO$), 0.87 (3H, s, $C(C_AH_3)(C_BH_3)$) and 0.77 (3H, s, $C(C_AH_3)(C_BH_3)$); δ_C ($CDCl_3$, 75 MHz) 171.6 (*C*=O), 136.3 (C_{ipso}), 128.2 ($C_{Ar}H$), 127.8 ($C_{Ar}H$), 127.5 ($C_{Ar}H$), 89.1 (1H, s, >*C*HO-), 34.2 (CH_2), 33.3 ($C(CH_3)_2$), 27.5 (CH_2), 26.8 ($C(C_AH_3)(C_BH_3)$) and 19.4 ($C(C_AH_3)(C_BH_3)$); *m/z* (CI+) 205.12 ($[M+H]^+$, 68%), 187.11 (100), 145.10 (80), 127.08 (39), 105.07 (30) and 91.05 (15); HRMS (CI+) 205.1237 ($[M+H]^+$), $[C_{13}H_{18}O]^+$ requires 205.1229. Enantioselectivity determined by chiral HPLC. Chiralpak AD, 1 mL/min, 95:5 hexane:2-propanol. Retention times 19.9 min (minor enantiomer), 28.0 min (major enantiomer). Data are in agreement with the literature for the racemic compound that has been reported before.[15]

References

1. I. P. Evans, A. Spencer, and G. Wilkinson, *J. Chem. Soc., Dalton Trans.*, 1973, 204-209.
2. (a) M. L. Clarke, M. B. Diaz-Valenzuela, A. M. Z. Slawin, *Organometallics*, 2007, **26**, 16-19. (b) S. D. Phillips, J. A. Fuentes, and M. L. Clarke, *Chem. Eur. J.,* 2010, 16, 8002
3. B. J. Barnes, P. J. Newcombe and R. K. Norris, *Aust. J. Chem.*, 1983, **36**, 963.
4. P. Wessig and O. Mühling, *Helv. Chim. Acta*, 2003, **86**, 865-893.
5. A. D. Campbell, C. L. Carter, and S. N. Slater, *J. Chem. Soc.*, 1948, 1741-1746.
6. R. Johnson and N. Riggs, *Aust. J. Chem.*, 1971, **24**, 1643-1658.
7. W. J. Li and S. X. Qiu, *Adv. Synth. Catal.*, 2010, **352**, 1119-1122
8. J. A. Fuentes, M. B. France, A. M. Z. Slawin, and M. L. Clarke, *New J. Chem.*, 2009, **33**, 466-470.
9. W. S. Trahanovsky and J. Cramer, *J. Org. Chem.*, 1971, **36**, 1890-1893.
10. S. Winstein and B. K. Morse, *J. Am. Chem. Soc.*, 1952, **74**, 1133-1139.
11. M. C. P. Yeh, P. Knochel, and L. E. Santa, *Tetrahedron Lett.*, 1988, **29**, 3887-3890.
12. R. Downham, P. J. Edwards, D. A. Entwistle, A. B. Hughes, K. S. Kim, and S. V. Ley, *Tetrahedron: Asymmetry*, 1995, **6**, 2403-2440.
13. T. Dohi, N. Takenaga, A. Goto, A. Maruyama, and Y. Kita, *Org. Lett.*, 2007, **9**, 3129-3132.
14. M. Newman and S. Gupte, *J. Org. Chem.*, 1970, **35**, 4176-4180.
15. R. Johnson and N. Riggs, *Aust. J. Chem.*, 1971, **24**, 1643-1658
